# Utilizing logistic regression to compare risk factors in disease modeling with imbalanced data: a case study in vitamin D and cancer incidence

**DOI:** 10.3389/fonc.2023.1227842

**Published:** 2023-09-28

**Authors:** Mohammad Meysami, Vijay Kumar, McKayah Pugh, Samuel Thomas Lowery, Shantanu Sur, Sumona Mondal, James M. Greene

**Affiliations:** ^1^ Department of Mathematics, Clarkson University, Potsdam, NY, United States; ^2^ Department of Mathematical Sciences, University of Northern Colorado, Greeley, CO, United States; ^3^ Department of Mathematics and Statistics, Slippery Rock University, Slippery Rock, PA, United States; ^4^ Department of Biology, Clarkson University, Potsdam, NY, United States

**Keywords:** 25-hydroxyvitamin D, cancer incidence, imbalanced data, randomized controlled trial, undersampling

## Abstract

Imbalanced data, a common challenge encountered in statistical analyses of clinical trial datasets and disease modeling, refers to the scenario where one class significantly outnumbers the other in a binary classification problem. This imbalance can lead to biased model performance, favoring the majority class, and affecting the understanding of the relative importance of predictive variables. Despite its prevalence, the existing literature lacks comprehensive studies that elucidate methodologies to handle imbalanced data effectively. In this study, we discuss the binary logistic model and its limitations when dealing with imbalanced data, as model performance tends to be biased towards the majority class. We propose a novel approach to addressing imbalanced data and apply it to publicly available data from the VITAL trial, a large-scale clinical trial that examines the effects of vitamin D and Omega-3 fatty acid to investigate the relationship between vitamin D and cancer incidence in sub-populations based on race/ethnicity and demographic factors such as body mass index (BMI), age, and sex. Our results demonstrate a significant improvement in model performance after our undersampling method is applied to the data set with respect to cancer incidence prediction. Both epidemiological and laboratory studies have suggested that vitamin D may lower the occurrence and death rate of cancer, but inconsistent and conflicting findings have been reported due to the difficulty of conducting large-scale clinical trials. We also utilize logistic regression within each ethnic sub-population to determine the impact of demographic factors on cancer incidence, with a particular focus on the role of vitamin D. This study provides a framework for using classification models to understand relative variable importance when dealing with imbalanced data.

## 1 Introduction

In public health, it is often of interest to understand the significance of explanatory variables with respect to a particular phenomenon. For example, one may wish to quantify the relative importance of smoking tobacco versus diet on cancer incidence, the likelihood of drug resistance as a function of chemotherapy protocol, or the significance of air pollution on infant mortality compared with socioeconomic status. These questions are often addressed by the analysis of large data sets, which are often collected from clinical trials or extended surveillance studies. One method incorporating such data that may be utilized to compare the contribution of explanatory variables is multivariate logistic regression. Indeed, the goal of regression is often twofold: to make accurate predictions for unobserved data, and to understand the extent to which each variable influences the prediction. When interested in the latter, there exist a number of techniques which can be utilized to assess the relative importance of predictor variables; for example, see Tonidandel and LeBreton (1).

An issue which frequently prevents the calibration of logistic regression models, and hence obstructs conclusions regarding variable importance, is that of *imbalanced* (also known as *unbalanced*) data sets, especially with regards to discrete classification problems (2–5); we note that methods to classify continuous target variables in imbalanced data sets are also being developed Yang et al. (6). Imbalanced data refers to a highly skewed data set, which for simplicity we assume can be partitioned into *majority* (non-event) and *minority* (event) classes with respect to the classifier label (i.e. we consider a discrete, binary classification problem). Imbalanced data sets are those for which the number of observations are heavily skewed towards the majority class, with a common benchmark being a two-to-one ratio or more for the majority class (3). When presented with imbalanced data, many classical statistical and machine learning methods fail to accurately identify the minority class, which is often the class of interest in biological applications. For example, ANOVA models inherently assume a balanced data set (7). More precisely, such methods often converge to models that are highly *accurate*, but have a very low *specificity*; note that specificity is often a more applicable metric when predicting and understanding disease prevalence.

As an example, consider the case of understanding the role of different explanatory variables in cancer incidence. Specifically, we may be interested in determining the relative influence of these variables on the probability of developing cancer by a certain age. Motivated by the discussion above, we construct a multivariate logistic binary classification model with outcomes corresponding to positive and negative cancer diagnoses, and explanatory variables corresponding to demographic and biometric data, such as age, sex, body mass index (BMI), and race. This model is then calibrated to publicly available surveillance data, such as the VITAL data set discussed below. However, such data will generally be highly imbalanced, with a majority of the data points corresponding to a negative cancer diagnosis for the duration of the surveillance period. Thus, classical model fitting techniques will heavily bias the model towards this majority class (a negative cancer diagnosis), and while being highly accurate, will be ineffective with regards to cancer diagnosis (true positives), and thus possess a sensitivity of approximately zero. Such a model thus provides no information with regards to explanatory variables, as an ideal cancer incident model should exhibit consistent performance across both majority and minority classes; it is from such a model that the relative importance of risk factors can be inferred. We note that despite significant research in cancer statistics, minimal work exists addressing the specific problem of imbalanced data in cancer data sets. Studies in fields such as ecology and credit scoring have shown that dealing with imbalanced data prior to fitting a model can improve model predictions for response variables (4, 5, 8).

To address the aforementioned issue of imbalanced data, several strategies exist. One of the most well understood methods is that of resampling the data sets to remove the disparity between the majority and minority class sample sizes. Two approaches are possible: *oversample* the minority class, or *undersample* the majority class. Note the goal of both of these strategies is to manipulate the original data set so that classical statistical techniques can be applied, as discussed previously. In general, such sampling is performed to maintain the original (i.e. marginal with respect to each class) distributional characteristics of both classes, so that the new data set is indeed representative of the original sample. Many techniques for oversampling exist, such as simple sample with replacement, or synthetic minority oversampling technique (SMOTE) (9), which creates new minority class samples from *k*-nearest neighbors. Similarly, undersampling the majority class can be performed in a number of ways, including a simple random sample from the majority class, or implementing heuristic *near-miss* rules for selecting the majority sub-sample (10). Other than data manipulation, other techniques for dealing with imbalanced data include modifying the loss function associated to the statistical model (11) and hybrid approaches that combine both data set and algorithmic approaches. For a more detailed discussion of such methods, as well as their relative merits, we refer the reader to Johnson and Khoshgoftaar (12) for a detailed review.

In this work, we propose a systematic method for comparing the relative importance of explanatory variables for large imbalanced data sets arising in public health. Specifically, we develop a method which combines a novel undersampling technique with binary logistic regression to determine relative importance of biometric and socioeconomic variables in disease incidence. We emphasize that our goal is not derive a fully predictive model, but rather to develop a framework which can be used to extricate the contributions of different confounding risk factors with respect to phenomena in biomedical sciences. We note that our method utilizes undersampling, as recent studies suggest that undersampling techniques may be more effective in addressing the skewness of a data set and can thus outperform oversampling techniques compared to oversampling techniques (13–15). Furthermore, undersampling techniques do not synthesize artificial data, but instead utilize only existing (i.e. real) values in the original data set. In this way, we view our approach as involving a minimal amount of data manipulation, and relies entirely on actual, as opposed to synthetic, data. As a case study, we apply our method to understand the role vitamin D plays in cancer incidence and prevention with respect to other classical risk factors, such as age, sex, BMI, as well as how the relative importance of these risk factors changes as a function of ethnicity.

### 1.1 Cancer and vitamin D

Cancer is a collection of genetic diseases which are characterized by uncontrolled cellular growth and the ability to metastasize to distal locations in the body (16, 17). In the United States of America, 1,918,030 new cancer cases and 609,360 cancer deaths are projected to occur in the year 2022, where it is the second leading cause of death (18); worldwide, it accounts for approximately one out of every six deaths every year (19). There are over 100 different types of cancer, which are generally characterized by the type of the cell where the disease initiates. The most common cancer sites include lung and bronchus, breast (women), prostate (men), colon and rectum, ovarian (women), lymph nodes, and skin (18). Extensive epidemiological data reveal that sex, ethnicity, and socioeconomic status substantially impact both cancer incidence and mortality rates (20).

Several potential risk factors for cancer, including tobacco use, obesity, and diet, have been identified through both epidemiological and experimental studies (21). Sex and age can also significantly impact an individual’s risk for various types of cancer, with sex playing a large role in many types of cancer and older age generally increasing the risk of a positive cancer diagnosis (22, 23). Additionally, race has been shown to impact cancer risk. For example, research has shown that, in breast cancer, the age of diagnosis was younger in nonwhite patients (24). While many risk factors have been identified, the relative importance and interaction of various risk factors have yet to be fully characterized, and remains one of the most important questions in cancer research (25). It is also known that external factors, such as diet, can considerably contribute to the development of cancer (26).

Vitamin D is a group of fat-soluble prohormones which assist the body in the utilization of calcium, phosphate, and magnesium (27). It appears both naturally and as an additive to some foods, is available as a dietary supplement, and can be synthesized endogenously via exposure to ultraviolet (UV) sunlight (28). Although primarily associated with the health of bones and teeth, many early (i.e. before 2004) epidemiological studies suggested low 25-hydroxyvitamin D [25(OH)D] concentration were positively associated with many types of cancer incidence, including colorectal, breast, ovarian, and prostate cancers (29, 30). Specifically, both incidence and death rates for certain cancers have been observed to be lower for equatorial locations which experience a higher concentration of UV radiation, leading many researchers to hypothesize that vitamin D concentrations may be causally linked to this association (29–31). There is also experimental evidence that vitamin D may negatively regulate cellular processes associated with carcinogenesis. For example, murine models have shown that vitamin D receptors reduce cell proliferation and differentiation (32, 33), and similar results have also been observed for colon cancer in humans (34). Anti-angiogentic properties of vitamin D have also been observed in cell culture and murine models (35).

Recent evidence has further suggested that vitamin D may play a role in cancer prevention and management. Such research efforts underscore the significance of continued investigations to elucidate the efficacy of vitamin D in reducing cancer risk and improving patient outcomes. For example, investigators in Zhou et al. (36) conducted a prospective evaluation, revealing an inverse linear relationship between 25-hydroxyvitamin D (25(OH)D) concentrations and colorectal cancer (CRC) risk. These findings align with Munoz and Grant (37), which highlighted ecological and observational evidence supporting vitamin D’s anticancer actions and a wide range of experimental studies describing various anticancer effects of vitamin D compounds. Further supporting the significance of vitamin D, Arayici et al. (38) utilized a meta-meta-analysis method to examine the effects of vitamin D intake and serum 25(OH)D concentrations on cancer incidence and mortality. Their findings concluded that increased vitamin D intake and serum 25(OH)D concentrations were associated with reduced cancer risk and mortality. Significantly, they emphasized the importance of evaluation based on specific cancer types. The association between 25(OH)D concentrations and cancer risk in individuals with metabolic syndrome was explored in (39), where the authors identified an inverse correlation between 25(OH)D concentrations and the risk of colon, lung, and kidney cancer, providing further support for the potential role of vitamin D in cancer prevention. Complementing these findings, Kuznia et al. (40) conducted a systematic review and meta-analysis of randomized controlled trials (RCTs) to assess the effect of vitamin D3 supplementation on cancer mortality. While the main meta-analysis of 14 RCTs did not demonstrate a statistically significant reduction in cancer mortality, subgroup analyses suggested a potential benefit with daily dosing of vitamin D3, particularly among adults aged at least seventy years and those initiating vitamin D3 therapy before cancer diagnosis. Additionally, it is known that clinical trials involving vitamin D ignore baseline concentrations of the subjects, which can have confounding impacts on the outcome (41–43). As an example, cancer may have been present but undiagnosed prior at the onset of the clinical trial, as well as the fact that it takes certain amount of time for vitamin D supplementation to increase serum 25(OH)D concentrations (44). For an extensive literature review on earlier epidemiological, clinical, and experimental data relating vitamin D and cancer, we refer the interested reader to (45).

Building on the foundational findings and collective research efforts regarding the associations between vitamin D and cancer risk and survival, in this work we analyze the VITamin D and OmegA-3 TriaL (VITAL) to investigate the relative importance of vitamin D in cancer incidence and mortality. The VITAL data was a clinical trial to provide a publicly-available data set for investigating the effects of vitamin D on disease incidence. Since its completion in 2018, a number of studies have been published analyzing the results of the VITAL data set; however, findings from such studies are often inconsistent and even conflicting due to the complexity and challenges of conducting large-scale clinical trials (46–49). Here, we are interested in understanding the role of vitamin D in cancer prevention. However, it is well known that the effect of vitamin D is highly variable. For examples, BMI plays a role in an individual’s response to vitamin D supplementation as a result of immune dysfunction (50, 51) due to increased systemic inflammation (52), vitamin D deficiency is more common among older men than in other populations (53), and black adults have a higher prevalence of vitamin D deficiency due to reduced skin vitamin D synthesis (54, 55). Leveraging data from the VITAL study, we will analyze how these variables individually influence cancer outcomes and explore the unique contribution of vitamin D in the context of cancer prevention and management. The goal of this work is to use our proposed framework to understand the *relative importance* of vitamin D in cancer incidence/mortality with respect to the other well-known cancer risk factors of ethnicity, BMI, age, smoking, and sex utilizing the data provided by the VITAL study.

## 2 Methods

The proposed framework for understanding the relative importance of predictors from clinical trial data can be summarized in the following three-step procedure:

Step 1: Undersampling
Step 2: Logistic regression
Step 3: Predictor importance

**Figure 1:**
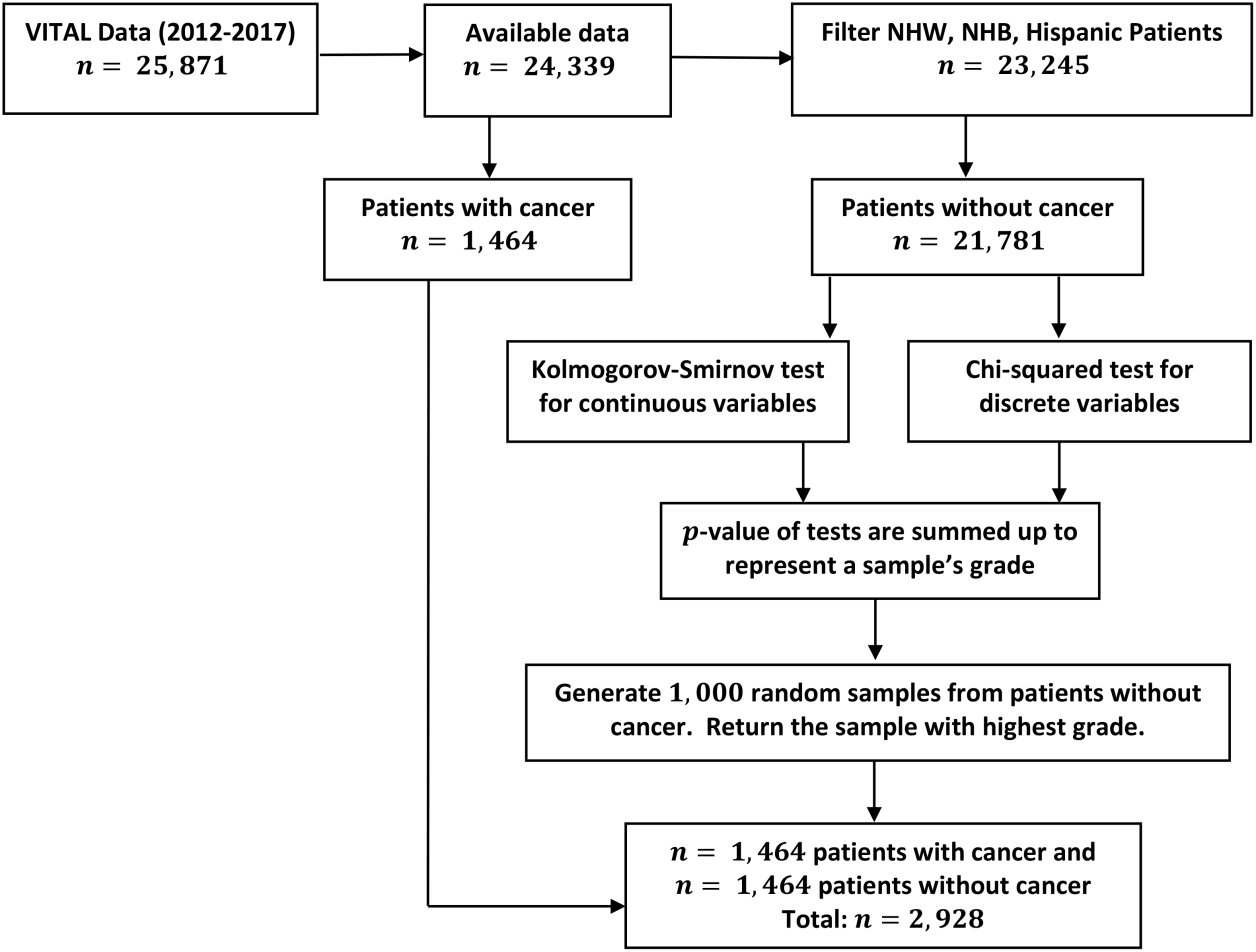
Outline of data manipulation performed in the analysis of the VITAL data set. Details on undersampling method (Step 1) performed to balance cancer diagnosis response variables.

In general, we begin with a highly imbalanced data set, which contains skewed data with respect to outcomes of interest (e.g. positive cancer diagnosis); see Sections 2.1- 2.3 for a discussion of the data analyzed in this manuscript, as well as pre-processing techniques and methods of quantification. In Step 1, we undersample the majority class to create a balanced data set. The proposed method of undersampling is novel, and is discussed in detail in Section 2.5 (see also **Figure 1**). Once a balanced and representative subsample is obtained, in Step 2 a multivariate logistic regression model is calibrated to the balanced data to understand disease outcome as a function of predictors of interest; see Section 2.4. Step 3 then involves ranking predictor importance with respect to outcome, as in (56, 57), and is discussed in Section 2.4.

**Figure 2:**
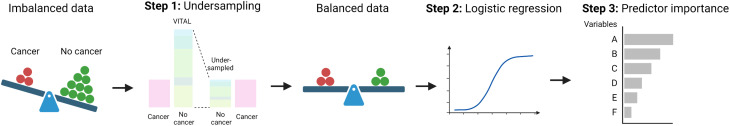
General procedure for determining the relative importance of risk factors in an imbalanced data set. For details on the undersampling technique (Step 1), see **Figure 1**.

A diagram depicting the above steps as a paradigm for determining the relative importance of predictors from imbalanced data sets is provided in **Figure 2**. We emphasize that although this method is applied to a study of the effect of vitamin D on cancer incidence and mortality, it is generally applicable to a wide array of diseases, where one is interested in understanding the relative importance of specific variables on a desired outcome.

### 2.1 Data collection

The VITAL trial aimed to evaluate the potential advantages of consuming daily supplements of Vitamin D3 (2000 IU) and omega-3 fatty acids in preventing cancer, heart disease, and stroke. The trial was designed as a randomized, double-blind, factorial study, so that individuals were randomly assigned to one of four groups: receiving vitamin D3, omega-3 fatty acids, a combination of both, or a placebo. Neither participants nor researchers were aware which treatment a participant received, so results of the study were as unbiased as possible. From a pool of 401,605 potential participants, 25,871 were chosen to take part in the 5-year intervention phase of the study, which ended on December 31, 2017. Participants were given a fresh supply of pills every year, along with follow-up inquiries about adherence, potential negative effects, and the occurrence of endpoints. The VITAL data was collected from men at least 60 years of age, and women at least 65 years of age. Participants were selected specifically who did not have a prior history of cancer and cardiovascular disease from the entirety of the United States. Men and women were selected in equal proportion, and the study included at least 5000 nonHispanic Black individuals, as this was a target demographic for understanding the role of vitamin D supplementation. Throughout the study, several clinically significant variables were monitored, such as the incidence of cancer and related mortality, the onset of diabetes, and the number of strokes. The main focus of the analysis presented here is on cancer diagnosis to gain better insight into the role of vitamin D on prevention. At the time of writing, the data used for this study can be accessed at https://data.projectdatasphere.org/projectdatasphere/html/access.

### 2.2 Data pre-processing

As discussed in the Introduction, cancer incidence and mortality rates vary greatly among different racial and ethnic groups. The primary focus of this study determining the relative importance of age, sex, BMI, smoking, and vitamin D intake in relation to cancer incidence in the non-Hispanic Black (NHB), non-Hispanic White (NHW), and Hispanic ethnic groups. For the remainder of the work, we refer to these individual characteristics as *variables*. Other ethnic groups, such as Asian (388 participants, 1.5% of total population), Native American (388 participants, 0.9% of total population), and other races (523 participants, 2% of total population) were not included in the analysis presented here due to the limited number of participants which prevented any statistically significant conclusions from being ascertained.

To investigate the distribution of each variable in each ethnic group, we define the *relative proportion* of variables in each ethnic group. For a given variable *i* with *k* distinct values and ethnic group *j*, the relative proportion of *i* in *j* at value *k* is formally defined as


$$RP_{\left(j,k\right)}=\frac{\#\text{of}\;\text{participants}\;\text{with}\;\text{variable}\;\text{value}\;i=k\;\text{in}\;\text{ethnic}\;\text{group}\;j}{\text{total}\;\#\;of\;\text{participants}\;\text{in}\;\text{variable}\;i\;\text{in}\;\text{ethnic}\;\text{group}\;j}$$

**Figure 3:**
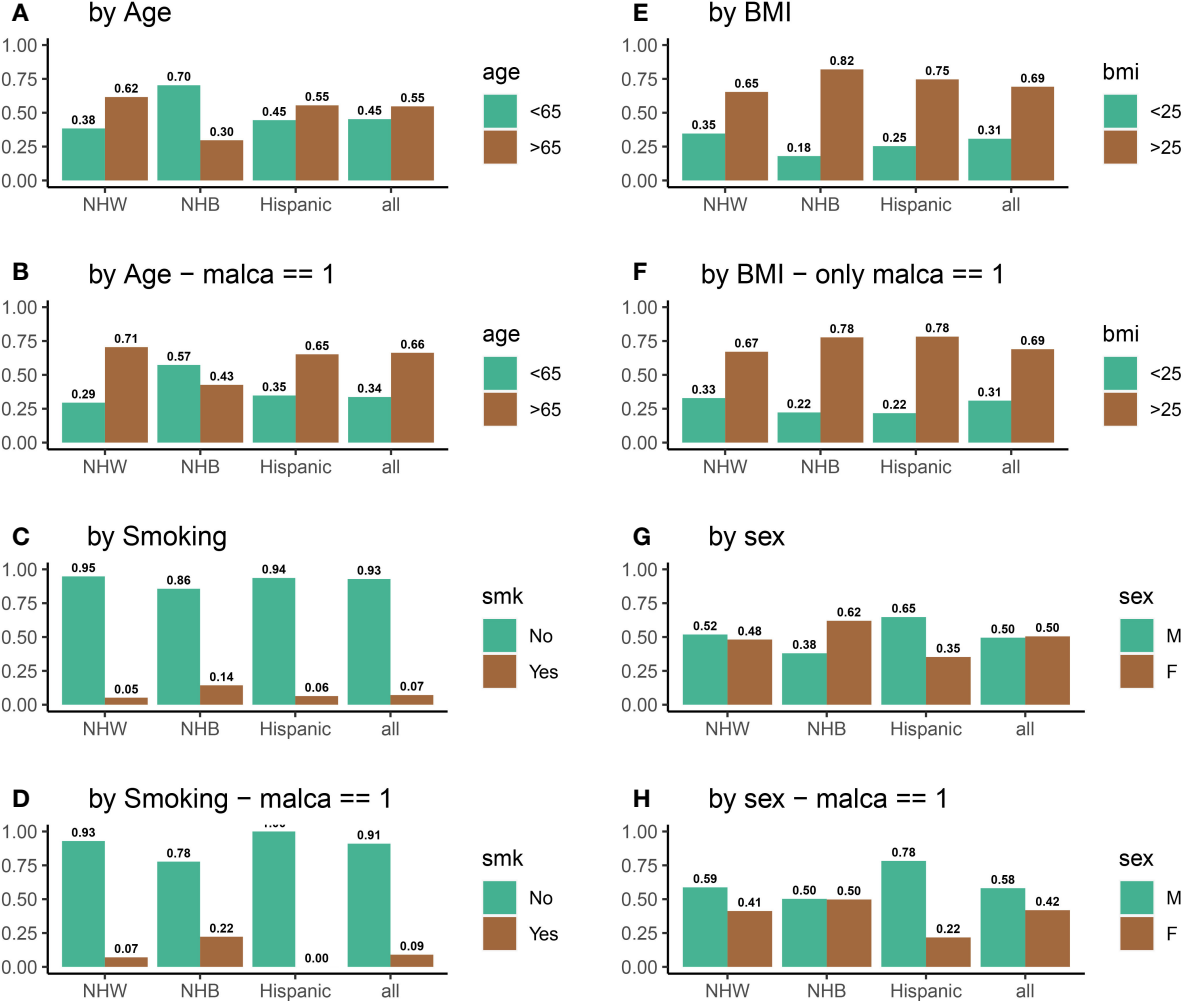
Distributions all of variables of interest for all participants **(A, C, E, G)** and for participants who were diagnosed with cancer **(B, D, F, H)** during the five-year VITAL study period.

The use of relative proportion allows us to compare the distribution of variable proportions between ethnic groups. As an example, consider the NHW ethnic group, i.e. let *j* = NHW. After removing missing values, there are a total number of 17,451 participants in the NHW ethnic group. Suppose that we are interested in the proportion of NHW individuals participating in the VITAL data set who smoke, so that we fix *i* = smoking. The variable *i* = smoking has two values, “Yes” and “No”, so that *k* ∈{smk=Yes, smk=No}. Among all NHW participants, 16,541 smoke, while the remaining 910 do not. Thus, the relative proportion for nonsmokers in the NHW ethic group is *RP*_(NHW, smk=No)_ = 16541*/*17451 = 0.95 and the relative proportion of those who smoke is *RP*_(NHW, smk=Yes)_ = 910*/*17451 = 0.05; this example is also visualized in **Figure 3C**. The relative proportion may be similarly calculated for all other variables among the NHW, NHB, and Hispanic ethnic groups.

### 2.3 Statistical analysis

#### 2.3.1 Data summary

Among the total 25,871 randomized participants, 71% were reported as NHW, 20% as NHB, 4% as Hispanic, with the remainder labeled as other or unclassified races. Males made up 12,786 of the participants, with a mean age of 65.6 years, and 13,085 were females with a mean age of 67.6 years. The mean age of all participants was 66.6 years. Among all participants, 7,843 possessed a BMI of at most 25, and 17,411 participants had a BMI higher than 25. **Table 1** summarizes screening, randomization, and patient characteristics. More details about the VITAL data set can be found in Manson et al. (58).

**Table 1:** Participant characteristics and basic summary statistics of the VITAL clinical trial evaluating the effects of vitamin D supplementation.

	# of participants	Age (± SD)	BMI (± SD)
Patients completed initial screening	401, 605		
Patients willing and eligible to participate	39, 430		
Patients did not adhere to trial regimen or became unwilling to participate	13, 559		
Randomized participants	25, 871		
Participants received active vitamin D	12, 927		
Participants received placebo vitamin D	12, 944		
Alive participants at end of intervention	24, 893	66.4 (59.5, 73.4)	28.1 (22.4, 33.7)
Dead participants at end of intervention	978	71.2 (62.3, 80.1)	28.6 (21.4, 35.8)
Males	12, 786	65.6 (58.4, 72.7)	27.8 (23.0, 32.5)
Females	13, 085	67.6 (60.8, 74.4)	28.4 (21.8, 34.9)
Age ≤ 65	11, 533	60.6 (56.8, 64.3)	28.9 (22.8, 35.1)
Age > 65	14, 338	71.5 (66.4, 76.6)	27.4 (22.1, 32.7)
BMI ≤ 25	7, 843	67.8 (60.6, 75.1)	22.6 (20.8, 24.4)
BMI > 25	17, 411	66.0 (59.2, 72.9)	30.6 (25.4, 35.7)
Smoking	1, 836	63.7 (57.2, 70.2)	27.8 (21.6, 34.1)
non-smoking	23, 649	66.8 (59.8, 73.9)	28.1 (22.4, 33.8)
NHW	18, 046	67.6 (60.8, 74.3)	27.4 (22.1, 32.6)
NHB	5, 106	62.8 (55.0, 69.7)	30.6 (23.8, 37.3)
Hispanic	1, 013	66.8 (60.2, 73.5)	28.6 (23.1, 34.2)
Vitamin D	12, 927	66.6 (59.6, 73.7)	28.1 (22.4, 33.8)
Placebo	12, 944	66.6 (59.6, 73.7)	28.1 (22.3, 33.8)

The table presents the number of participants at each stage, including initial screening, eligibility, randomization, and intervention assignment. The summary statistics include age (mean ± standard deviation) and BMI (mean ± standard deviation) for various subgroups. The trial enrolled a total of 401,605 participants, and 25,871 were chosen to take part in the 5-year intervention phase of the study, which ended on December 31, 2017.

### 2.4 Logistic regression

To investigate the association between invasive cancer (of any type) and individual variables, a multivariate logistic regression (59) was utilized. Logistic regression is a statistical technique utilized to examine the relationship between a binary outcome (in this case, the presence or absence of a positive cancer diagnosis) and one or more independent variables (here age, sex, BMI, ethnicity, smoking status, and vitamin D protocol). The logistic regression model outputs the probability of an individual experiencing a particular outcome, as a function of the patient variables. These variables can be either discrete (e.g. categorical variables) or continuous (e.g. BMI). Logistic regression is widely used in the medical field to estimate the likelihood of developing diseases, such as diabetes, heart disease, or cancer, based on patient data (60, 61).

We aim to understand the relationship between various characteristics and the likelihood of developing cancer by using logistic regression. The outcome variable in this analysis is binary, indicating whether or not a participant developed cancer during the study. The predictor variables we are considering include age, BMI, sex, vitamin D or placebo use, and smoking status. By analyzing these variables, we seek to identify which factors may increase or decrease the risk of cancer and develop strategies for prevention or early detection. The objective of this study is not to predict cancer, but rather to investigate the connections between inter-person explanatory variables and cancer, and to assess the degree to which these characteristics may play a role in causing cancer. To evaluate the importance of each characteristic in this context, we used a model-based method for calculating variable importance, as described in (56, 57). Briefly, this method ranks predictors based on standard deviations of the partial-dependence plots (PDPs), which serve as an indicator of “flatness” of PDPs; a greater degree of “flatness” in PDPs implies less influence on the response variable. This ranking then allows us to determine the degree to which predictor variable is associated with a positive cancer diagnosis in the logistic model.

Since most of individuals in the VITAL data set were not diagnosed with cancer (21,781*/*23,245 = 93.7%), a logistic regression model calibrated to the entire data set will be biased towards this majority class. In order to utilize the logistic regression model to understand the role of vitamin D with respect to other risk factors (i.e. variables), we developed a novel undersampling method to correct for this imbalance prior to fitting the logistic model. As there existed 1,464 positive cancer diagnosis in the three ethnic groups of interest (NHW, NHB, and Hispanic), we first undersampled the negative cancer diagnosis class (21,781 individuals) as described in Section 2.5, and then fit the binary logistic regression to this sub-sampled data set.

### 2.5 A novel undersampling method

To prevent bias in the logistic regression model towards the majority class (negative cancer diagnosis), we generated a sub-sample of this class equal in size to the number of cancer-diagnosed participants (1,464). That is, we undersampled the negative cancer diagnosis class so that both cancer and non-cancer outcomes were represented equally in the data set which was subsequently utilized to fit the logistic regression model. Two approaches were employed for undersampling, each with a goal of maintaining the distributional properties of the negative cancer diagnosis class. The first method aimed to reflect the distribution of the non-cancer population and involved taking 1,000 random samples, each of 1,464 individuals without replacement, from the majority class. To determine if the sample of non-cancer participants accurately reflected the distribution of the non-cancer population, we applied chi-squared and Kolmogorov-Smirnov tests to compare the sample’s distribution of all variables of interest (BMI, sex, treatment arm, current smoking, and age) with that of the population. If the *p*-value of these tests is high, it suggests that there is not a significant difference between the sample and the population with respect to this variable. For each variable, we thus obtain a *p*-value, which measures (inversely) the discrepancy of the distribution of the sample with respect to the population. From 1,000 random samples, we then select the one with the highest summed *p*-values, as it is the most similar to the population in terms of all of the variables tested. The algorithm for balancing non-cancer and cancer populations is shown in **Figure 1**.

The second method is similar, except that it selects non-cancer participants to control for age and sex by ensuring that the age and sex distribution of the sub-sample matches that of the cancer-positive population. This approach allows the impact of other factors on the outcome to be observed more clearly. To balance the age and sex distribution of the cancer population, the data were divided into subgroups based on age and sex, and random samples were picked from each subgroup as discussed in the previous paragraph.

### 2.6 Software

The statistical software R version 4.1.2 was used for analyzing the data and utilizing the methods, along with the following libraries in our coding: readxl, dplyr, tidyr, ggplot2, viridis, vip.

## 3 Results

### 3.1 Statistical analysis

**Figure 4:**
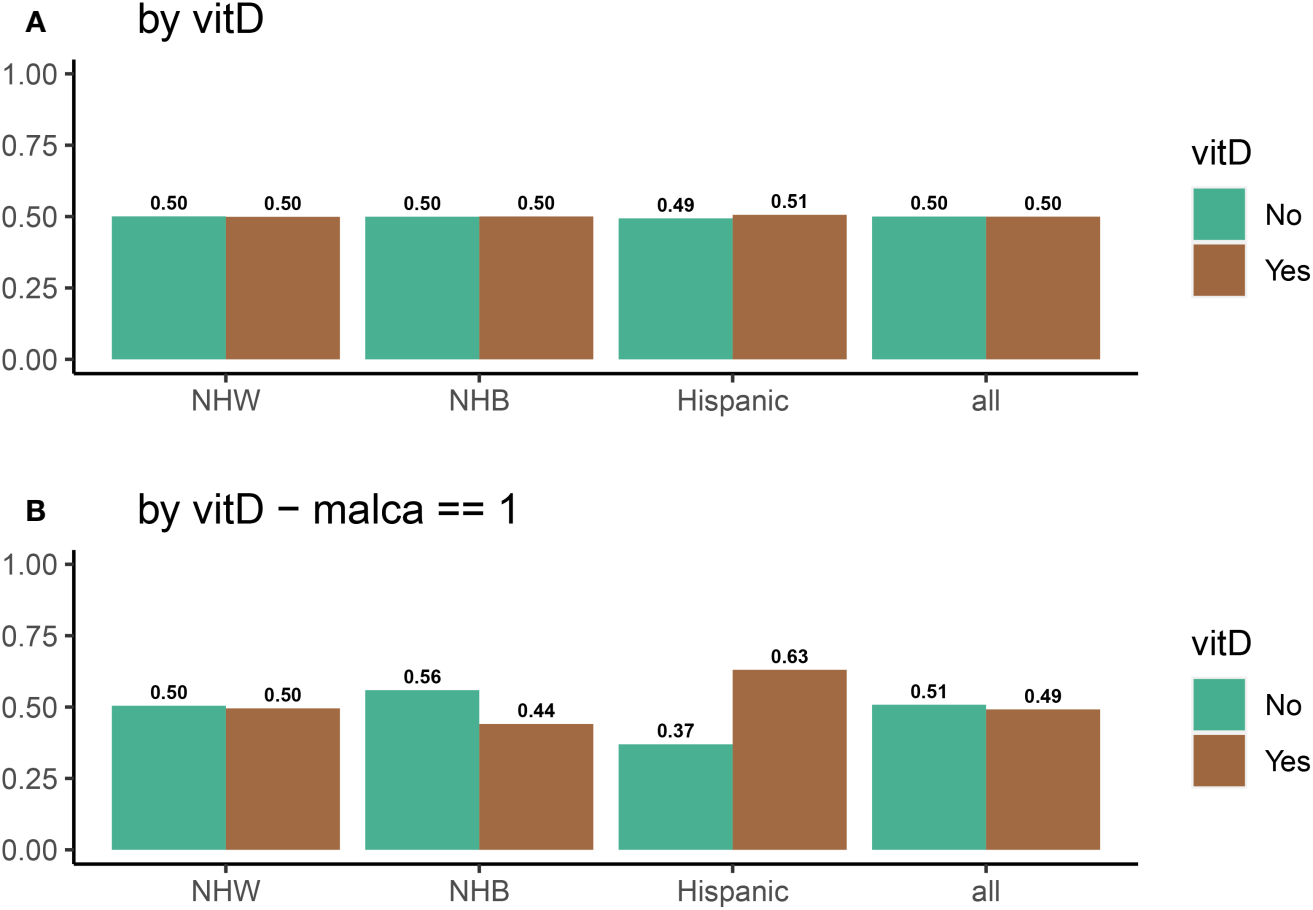
Distribution of vitamin D and placebo as a function of a race for **(A)** all participants, and **(B)** for participants who were diagnosed with cancer at some point during the VITAL study.

To investigate the impact of vitamin D on cancer incidence, data was analyzed from 25,871 participants in the VITAL study. **Figure 4A** shows that the distribution of individuals receiving vitamin D and placebo was identical among all ethnic groups. **Figure 4B** displays the vitamin D intake of participants who were diagnosed with cancer during the 5-year period of the study. There are substantial disparities between the vitamin D and placebo groups for NHB participants (VitD-proportion = 0.56, placebo-proportion = 0.44, *p* = 0.02) and Hispanic participants (VitD-proportion = 0.37, placebo-proportion = 0.63, *p* = 0.02). However, there is no significant difference between NHW participants who received a positive cancer diagnosis (VitD-proportion = 0.495, placebo-proportion = 0.505, *p* = 0.68) during this 5-year period. From this, we may initially conclude that vitamin D is effective in reducing cancer incidence among NHB participants, but not among NHW or Hispanic participants. However, by not considering the influence of other confounding variables, such as age, BMI, sex, and/or smoking which are known to have a significant impact in cancer incidence, it is difficult to infer the causal effect of vitamin D directly (62–65). For example, endometrial cancer has been linked with decreased age of diagnosis in obese individuals (64). Similarly, the risk of lung cancer is higher in NHB individuals who smoke (62, 63), and studies on breast cancer have revealed that age and BMI are important factors in cancer incidence rates (65). Thus, we examined the impact of vitamin D on cancer incidence in the context of other risk factors (variables), instead of concentrating solely on vitamin D intake. For example, compared to NHW and Hispanic participants, a higher percentage of NHB participants have a BMI above 25 (85% for NHB, compared to 65% and 75% for NHW and Hispanic, respectively) and smoke (14% for NHB, compared to 5% and 6% for NHW and Hispanic, respectively); the NHB ethnic group also has a younger (70% below 65 years old for NHB, compared to 38% and 45% for NHW and Hispanic, respectively) and more female population (62% for NHB, compared to 48% and 35% for NHW and Hispanic, respectively). Biologically, a high BMI and high smoking rate will increase the risk of cancer in the NHB group, while a higher proportion of younger females is expected to decrease this risk. The variation in risk factors between ethnic groups thus obscures any clear conclusions that can be made with respect to vitamin D and cancer prevention, and a more detailed analysis is required beyond descriptive statistics. Thus, we develop a systematic method for quantifying the relative importance of risk factors, as outlined in Section 2 (Steps 1 -3). For a complete description of variable proportions for the ethnic groups of interest, see **Figure 3**.

#### 3.1.1 Distributional differences in positive cancer diagnosis individuals

To quantify the previous statement regarding statistical differences in risk factors between ethnic groups for cancer positive individuals, we perform proportion tests; **Table 2** summarizes the results of these proportion tests for each variable of interest. In the NHW ethnic group, 38% of all participants were under 65 years old and 29% of participants who developed cancer were also under 65. The first row of **Table 2** indicates that the 95% confidence interval for the difference between the two proportions is (0.06,0.12) and the chi-squared test statistic value of 37.7, with a *p*-value of less than 0.001, confirms that the difference between 38% and 29% is statistically significant. **Table 2** demonstrates that there are significant proportion differences for age, smoking, and sex in both NHW and NHB groups between the entire data set and the positive cancer diagnosis (minority) class (*p <* 0.001, *p <* 0.01, *p <* 0.001 respectively). However, no proportion differences are statistically significant for the Hispanic group, which could be due to the small sample size (110 observations with a positive cancer diagnosis). We note that in the Hispanic sample, sex has the highest test statistic value and the smallest *p*-value.

**Table 2:** Proportion test results, as well as the 95% confidence interval, for each risk factor within each ethnic group.

	NHW		NHB		Hispanic	
	95% CI	Chi-squared	95% CI	Chi-squared	95% CI	Chi-squared
Age	(0.06, 0.12)	37.7***	(0.06, 0.20)	15.5***	(-0.06, 0.25)	1.3
Smoking	(0.00, 0.03)	7.1**	(0.02, 0.14)	9.5**	(-0.09, -0.04)	2.1
BMI	(-0.01, 0.04)	1.4	(-0.10, 0.02)	2.2	(-0.10, 0.17)	0.1
Sex	(-0.10, -0.04)	21.1***	(-0.19, -0.05)	12.3***	(-0.27, -0.00)	2.9
VitD	(-0.03, 0.03)	0.05	(-0.13, 0.01)	2.6	(-0.03, 0.28)	2.2

***p<0.001; **p<0.01; *p<0.05.

Here we analyze the difference between the distributional differences between the entire VITAL population data set and the subset with a positive cancer diagnosis in each variable (row) and in each ethnic group (column). The results here suggest that there are significant statistical differences between a number of predictive variables (including, but not limited to only vitamin D) in the cancer positive individuals. We thus apply the method outlined in **Figure 2** to understand the relative importance of predictors to quantify their importance in cancer diagnosis.

### 3.2 Logistic regression

To quantify how successful the logistic regression model is with respect to predicting cancer incidence, various performance measures may be utilized, as discussed in Section 1. Common metrics include measures of sensitivity (the probability that the test will identify the disease, assuming the patient does have the disease), specificity (the probability that the test will indicate the absence of the disease, assuming the patient does not have the disease), precision (the probability that the patient has the disease, given that the test has identified the presence of the disease), negative predictive value (NPV; the probability that a person who tests negative for the disease does not have the disease), and accuracy (probability that the test correctly detect patients with and without disease). Ideally, it would be desirable to have all these measures equal to one; however this is generally not achievable, particularly in cancer prediction where there are various latent and immeasurable factors. With respect to cancer prediction, a critical metric is that of sensitivity, i.e. a model with low sensitivity will generally not be scientifically useful.

Initially, we applied logistic regression to each of the three ethnic groups, utilizing cancer incidence as the response variable; recall that data is heavily imbalanced, with the majority class (no cancer diagnosis) containing approximately 93.7% of the data. The aim was to assess the model’s ability to accurately predict cancer incidence based on the available predictors. Furthermore, once the model is successfully calibrated to each ethnic group, our goal is to compare the relative importance of each risk factor between ethnic groups as discussed in Section 2.4 and more broadly in **Figure 2**. More specifically, we want to understand the variation in the efficacy of vitamin D as a cancer prophylactic as a function of ethnicity. The prediction metrics of the model are presented in **Table 3**. The findings show that the model (fit to the imbalanced data set) had a sensitivity of zero, a specificity of one, and an undefined precision, as the model predicts that no individual will develop cancer during the study. Note that despite the high degree of accuracy (between 93% and 96%), the model’s predictions with respect to those patients developing cancer were extremely poor, so that the model could not be used to provide information with respect to the explanatory variables relating to cancer incidence.

**Table 3:** Performance measures of logistic regression on full (imbalanced) VITA data set.

	Sensitivity	Specificity	Precision	NPV	Accuracy
All	0.00	1.00	NA	0.94	0.94
NHW	0.00	1.00	NA	0.93	0.93
NHB	0.00	1.00	NA	0.96	0.96
Hispanic	0.00	1.00	NA	0.95	0.95

To address this issue, we employed the undersampling technique as outlined in Section 2.5 (Step 1); distributional properties of the obtained sample are shown in (a) and (b) of **Figure 1**, and are provided to verify that it does indeed form a representative statistical sample of the entire cancer negative (majority) class. Utilizing this sample, we then performed the logistic regression analysis on the undersample, together with the positive cancer diagnosis groups (minority class), and obtained generally improved prediction metrics (Step 2). Specifically, we observed a significant improvement in sensitivity and positive predictive value across all groups. The sensitivity improved to 60% for all participants, 69% for NHW, 24% for NHB, and 26% for the Hispanic group. Similarly, the precision also improved to 57% for all groups, 58% for NHW, 58% for NHB, and 48% for the Hispanic group. This suggests that to obtain a logistic regression model applicable for disease instance, we must first balance the majority and minority classes. These findings have implications for cancer research and public health policy, as accurate prediction of cancer incidence can aid in early detection and prevention of the disease. A summary of all performance measurements for the balanced data is presented in **Table 4**.

**Table 4:** Performance measures of logistic regression (Step 2) on undersampled VITA data set.

	Sensitivity	Specificity	Precision	NPV	Accuracy
All	0.60	0.56	0.57	0.58	0.58
NHW	0.69	0.43	0.58	0.55	0.57
NHB	0.24	0.89	0.58	0.65	0.64
Hispanic	0.26	0.80	0.48	0.60	0.57

Compare to results in **Table 3**.

After obtaining a representative sample for the negative cancer diagnosis class and demonstrating that logistic regression can be utilized as a tool for understanding variable importance in cancer diagnosis, a more detailed logistic regression analysis is performed; a summary of is provided in **Table 5**. In the first column (labeled “All”), age, sex, BMI, smoking, vitamin D, and race (ethnicity) are used as explanatory variables. Note that here all ethnic groups are combined in the initial model, as we want to understand if ethnicity has any explanatory effect in cancer diagnosis. We find that sex (*p <* 0.001), age (*p <* 0.001), smoking (*p <* 0.01), and race (*p <* 0.05) are significant, however, BMI and vitamin D are not significant (*p* ≥ 0.05). Since the variables “raceNHW” and “raceHispanic” are both significant, this suggest that additional analysis should be performed to examine the overall effect of race. We utilized the Wald test to assess the significance of the three levels of race (NHW, NHB, Hispanic). The Wald test with output *χ* ^2 =^ 22, df = 2, *p <* 0.05 indicates that the effect of race is indeed significant. To further examine the significance of other variables within each racial group, we filtered the balanced data by race and conducted logistic regression analyses for each race as in **Table 4**. The variables age, sex, and smoking were found to be significant in the NHW group, with *p*-values less than 0.001, 0.001, and 0.01 respectively. Similarly, age, sex, and smoking were significant in the NHB group with *p*-values of less than 0.01, 0.05, and 0.05 respectively. However, none of the variables were found to be significant in the Hispanic group due to the small number of positive cancer diagnosis cases.

**Table 5:** Logistic regression summary for all participants, and for NHW, NHB, and Hispanic participants.

	All	NHW	NHB	Hispanic
age>65	0.5*** (0.08)	0.49*** (0.09)	0.61** (0.19)	0.47 (0.45)
sexF	−0.43∗∗∗ (0.08)	−0.40*** (0.09)	−0.44* (0.18)	-0.85 (0.47)
bmi>25	0.09	0.13	−0.20	0.28
	(0.08)	(0.09)	(0.23)	(0.49)
vitDYes	−0.05	−0.05	−0.09	0.57
	(0.08)	(0.09)	(0.18)	(0.41)
smkYes	0.47*** (0.14)	0.49** (0.19)	−0.46* (0.23)	-14.64 (1030)
raceNHB	−0.44*** (0.11)			
raceHispanic	−0.48* (0.20)			
AIC	3960	3110	713	153
BIC	4010	3140	739	169
Log Likelihood	−1970	−1550	−350	-71
Deviance	3950	3100	701	141
Num. obs.	2930	2280	542	110

∗∗∗p < 0.001; ∗∗p < 0.01; ∗p < 0.05

Numbers in parenthesis represent standard errors for each coefficient. This summary is based on the balanced data set including 2,928 participants (equal between positive and negative cancer diagnosis).

**Figure 5:**
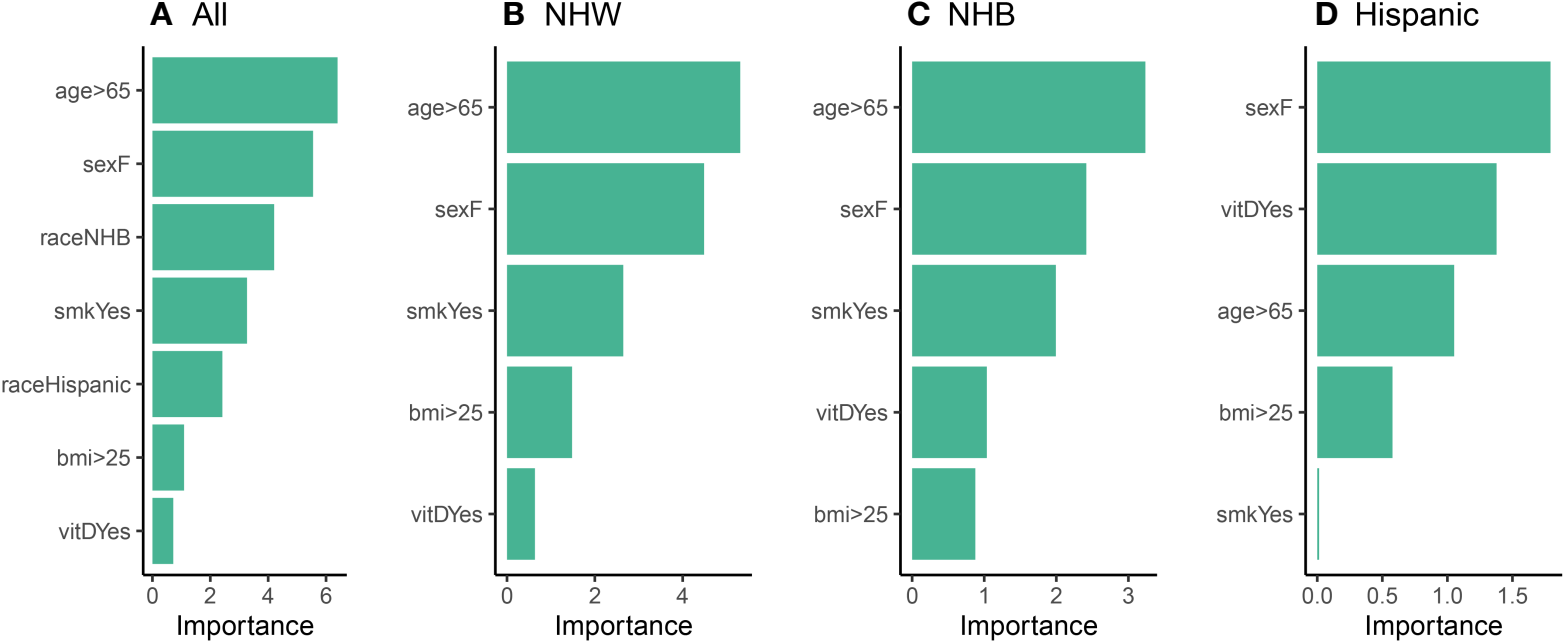
Relative variable importance plots (Step 3) for the logistic model using **(A)** all participants, **(B)** NHW participants, **(C)** NHB participants, and **(D)** Hispanic participants in the balanced data set.

We then utilized the logistic regression models to assess the relative variable importance of each variable (risk factor) in our study (Step 3). The results of this analysis are illustrated in **Figure 5**, where age and sex were identified as the most significant contributing factors for the “All”, NHW, and NHB models. When race was disregarded in **Figure 5A**, smoking emerged as the third most important factor across all three models. We note that vitamin D intake held the lowest importance rank in the “All” and NHW models, while in the NHB model, it held the second-lowest rank (above BMI). That is, it appears that vitamin D does play a role in cancer incidence for the NHB population, although it is not as significant as more classical risk factors such as age, sex, and smoking. Moreover, the importance of BMI and vitamin D were found to be relatively small and similar in the NHB group. Overall, these findings suggest that age, sex, and smoking are important factors to consider when studying the human characteristics that may influence our health outcomes. Nonetheless, additional research is necessary to understand the complex interplay between these variables and their impact on cancer incidence.

### 3.3 Comparison of undersampling with SMOTE

**Figure 6:**
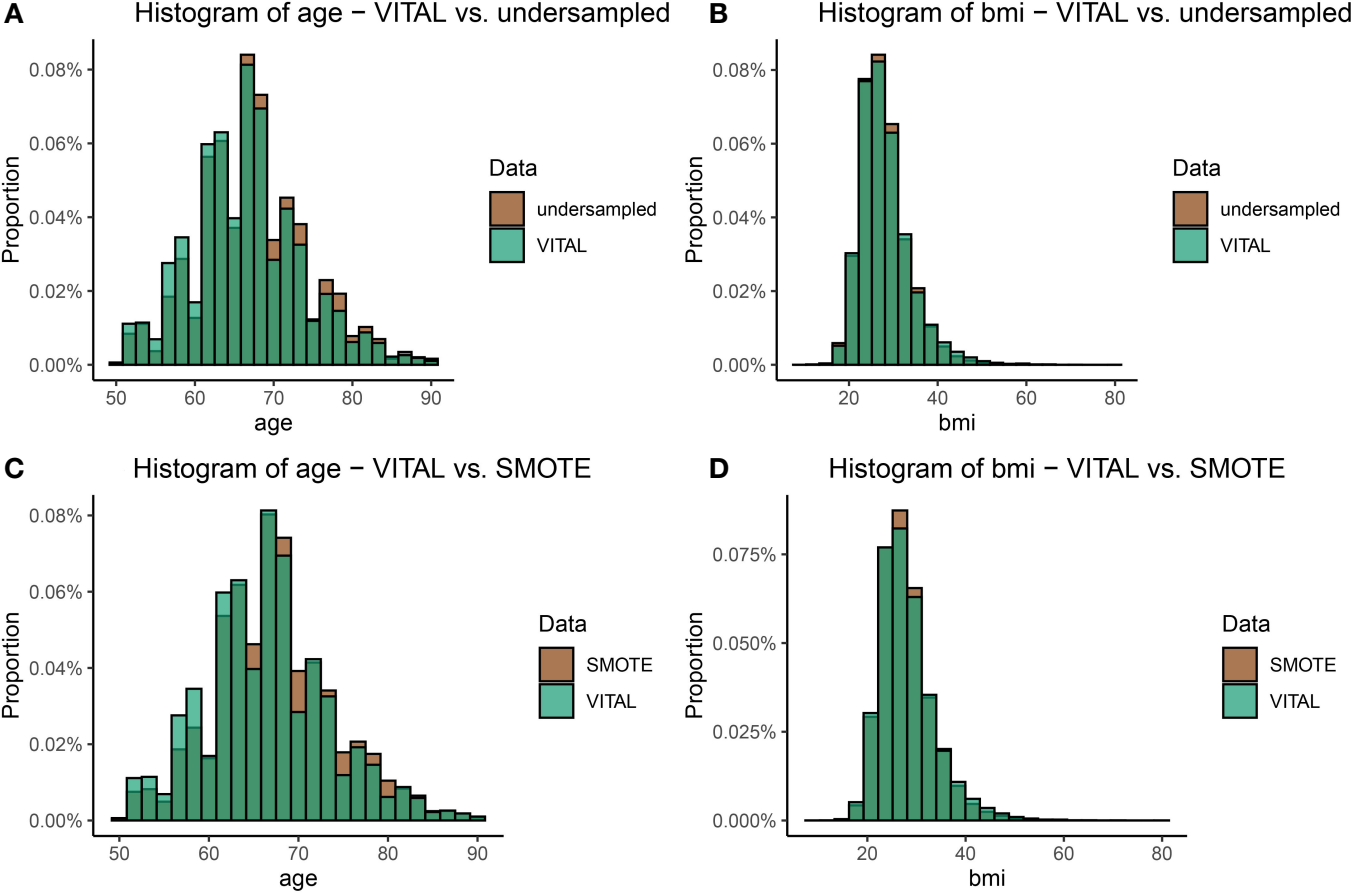
A visual comparison of the age and BMI distributions between the original VITAL dataset and the transformed datasets, which include the upsampled SMOTE dataset, and the undersampling technique outlined in Section 2.5 (Step 1). Plot **(A)** shows the comparison of the distribution of age in the original VITAL data set compared to the undersampled data. Plot **(B)** makes the comparison for BMI in the original VITAL data set compared to the undersampled data. Plots **(C)** and **(D)** make the same comparisons, as **(A)** and **(B)** respectively, but for the VITAL data and SMOTE.

We provide a comparison of our undersampling technique introduced in Section 2.5 to a standard oversampling technique, Synthetic Minority Oversampling Technique (SMOTE) (9). SMOTE works by generating new samples from the minority class by selecting a *k*-nearest neighbor in feature (variable) space, and then using a convex combination of features to generate additional samples; it is one of the most popular methods for handling class imbalance in data science and machine learning applications (66). To validate the distributional properties of the sampled data presented in Section 3.2 and investigate how well the undersampled data agreed with the SMOTE oversample, we compared the distribution of the undersampled data with the balanced SMOTE data. Results are provided in **Figures 6C, D**, and demonstrate that both SMOTE and the undersampling method exhibited distributions consistent with the original VITAL data, effectively achieving class balance. However, the key distinction lies in the fact that we the proposed undersampling technique utilizes only a subset of authentic data, avoiding the generation of any synthetic instances. This aspect highlights the strength and appeal of the undersampling method, as it maintains the overall distribution of the original dataset while adhering to the principle of using real data for model validation and inference.

## 4 Discussion and conclusion

Imbalanced data is a frequent problem that occurs in many applications, including disease modeling. In this work, we utilized the VITAL data as a case study to test our method of balancing the data together with logistic regression to understand the relative importance of classical risk factors in cancer incidence. To overcome biases associated with imbalanced data, we developed a novel undersampling technique, which we applied to the VITAL data to balance the majority and minority classes. We also compared the sample’s distribution of BMI, sex, current smoking, and age with that of the population using the Chi-square and Kolmogorov-Smirnov tests to ensure that the sample was a fair representation of the population. Our study found that balancing data is crucial in the development of accurate models for the prediction of cancer incidents.

Moreover, we used various evaluation metrics to assess the performance of our model before and after balancing the data. The results showed that balancing the data led to a significant improvement in the model’s performance. The model trained on the undersampled data had a higher sensitivity, precision, and balanced specificity compared to the imbalanced data models. We also tested our model on an independent data set to validate its performance, and the results were consistent with the training data.

By utilizing logistic regression analysis, we examined the confounding relationships between human risk factors and cancer incidence. Specifically, the goal of this work it to introduce a “pipeline” by which clinical data can be used to extrapolate the relative risk factors on disease incidence. A primary question of interest is how effective a treatment is, when compared to other risk factors inherit in the data set. We note that the answer to such a question is not immediately clear from descriptive statistics alone, as clinical data sets often present non-equivalent distributions with respect to risk factors between sub-populations.

Using VITAL data as a case study, we investigated the impact of vitamin D on reducing cancer risk among three different ethnic groups. Previous research (e.g. Sakamoto et al. (54)) has shown that the NHB populations tends to have lower vitamin D levels, which may increase their risk of cancer. It is also important to note that NHB individuals are more likely to have other risk factors for cancer, such as obesity and smoking, which may also contribute to their higher cancer risk. We examined the association between vitamin D intake and cancer incidence and how it may be affected by other risk factors. We specifically took into account the potential confounding variables of age, BMI, smoking, and sex.

Exploratory data analysis of the VITAL data set reveals mixed results on the relationship between vitamin D intake and cancer incidence. This could be due to complex interactions among human characteristics or a lack of significant association between vitamin D and cancer incidence. Though we found positive correlation between vitamin D intake and cancer incidence in NHB individuals, a closer examination revealed that the majority of the NHB sample was under the age of 65, while the majority of individuals in other ethnic groups were older. The same situation can be observed with respect to sex. According to the National Cancer Institute (NCI), men have a 50% chance of getting cancer during their lifetimes, while for women, the chance is around 33% Kim et al. (67). In this study, the majority of NHB participants were female, which may have contributed to a lower likelihood of cancer diagnosis among the NHB group. This raises doubts whether the observed cancer prevention in the NHB group is a result of vitamin D intake or simply a result of the majority of participants being female and younger. Furthermore, it should be noted that the VITAL clinical trial did not consider basal vitamin D levels and BMI, which could have contributed to the variation in cancer incidence (68–70).

Using our proposed statistical framework, the results of our analysis did not reveal a significant association between vitamin D intake and cancer incidence, at least with respect to classical risk factors. We were particularly interested in the role of vitamin D with respect to the NHB ethnic group, and although vitamin D did have an increased relative importance compared to the NHW population, it was still not as significant as age or sex. Additionally, the analysis revealed that the relationship between vitamin D intake and cancer incidence was not independent of other factors. Our findings did not support the notion that individuals with higher vitamin D intake have a lower risk of cancer compared to those with lower vitamin D intake when other human characteristics were taken into account. These results were consistent when race was considered as a factor and when each ethnic group was analyzed separately.

In conclusion, our suggested framework provides a modeling approach for understand the relative importance of risk factors in clinical data sets. With respect to vitamin D and the VITAL data set, it appears that although vitamin D could play a significant role in maintaining overall health by helping the body absorb and retain calcium and phosphorus, it does not play a significant role in cancer prevention. The inconsistent results from previous studies emphasize the need for more research to clarify the relationship between vitamin D and cancer incidence. To fully understand this relationship, it is essential to conduct further studies using large, diverse samples and considering the potential interactions and causality between vitamin D and other confounding factors.

## Data availability statement

Publicly available datasets were analyzed in this study. This data can be found here: https://data.projectdatasphere.org/.

## Ethics statement

Ethical approval was not required for the study involving humans in accordance with the local legislation and institutional requirements. Written informed consent to participate in this study was not required from the participants or the participants’ legal guardians/next of kin in accordance with the national legislation and the institutional requirements.

## Author contributions

MM: writing, coding, review, editing, conceptualization. VK: review, conceptualization, methodology, editing, investigation, coding, analysis. MP: Writing, data curation, visualization, formal analysis. SL: Data curation, visualization, formal analysis. SS: conceptualization, validation, editing. SM: supervision, conceptualization, validation, editing. JG: writing, review, conceptualization, editing, methodology, formal analysis, project administration. All authors contributed to the article and approved the submitted version.
